# MicroRNAs with prognostic significance in osteosarcoma: a systemic review and meta-analysis

**DOI:** 10.18632/oncotarget.19009

**Published:** 2017-07-05

**Authors:** Dong Cheng, Xubin Qiu, Ming Zhuang, Chenlei Zhu, Hongjun Zou, Zhiwei Liu

**Affiliations:** ^1^ Department of Orthopedics, The Third Affiliated Hospital of Soochow University, Changzhou 213003, P.R. China

**Keywords:** microRNAs, osteosarcoma, prognosis

## Abstract

**Introduction:**

This study aimed to elucidate the prognostic value of microRNAs (miRNAs) in patients with osteosarcoma.

**Materials and Methods:**

Studies were recruited by searching PubMed, Embase, the Cochrane Library, China National Knowledge Infrastructure, and Wanfang data-bases (final search update conducted January 2017). Eligible studies were identified and the quality was assessed using multiple search strategies.

**Results:**

A total of 55 articles that investigated the correlation between miRNA expression and either patient survival or disease recurrence in osteosarcoma was initially identified. Among these, 30 studies were included in the meta-analysis. The results of our meta-analysis revealed that elevated levels of miR-21, miR-214, miR-29, miR-9 and miR-148a were associated with poor prognosis in osteosarcoma. Additionally, downregulated miR-382, miR26a, miR-126, miR-195 and miR-124 expression indicated poor prognosis in osteosarcoma.

**Conclusions:**

miRNAs may act as independent prognostic factors in patients with osteosarcoma and are useful in stratifying risk.

## INTRODUCTION

Osteosarcoma is the most common bone malignancy with an incidence of 4–5 cases per million people [1]. It usually develops in the proximal humerus, proximal tibia, and metaphyseal regions of the distal femur. The probability of osteosarcoma occurrence is two times higher in boys than in girls. With the development of multidisciplinary treatments, the five-year survival rate has significantly improved to approximately 60–70% in patients with localized tumor. However, it is difficult diagnose osteosarcoma during the early disease stages, and approximately half of all patients develop metastases. These patients with metastasis or recurrence have a low 5-year survival rate [2]. Therefore, new biomarkers are essential to properly assess the prognosis of osteosarcoma. The biomarkers with most potential as prognostic factors are the microRNAs (miRNAs).

miRNAs are a class of endogenously expressed small non-coding RNAs 18–25 nucleotides in length. They negatively regulate gene expression by base pairing to the 3′ untranslated region of target mRNAs. These molecules are involved in almost all biological processes. Accumulating evidence suggests that miRNAs can function as tumor suppressors or oncogenes by targeting genes involved in tumor cell differentiation, proliferation, apoptosis and metastasis. Recently, many studies have shown that numerous miRNAs are either overexpressed or underexpressed in osteosarcoma [1] and are often associated with the entire process of tumor development [3]. The relationship between miRNA expression and the prognosis of patients with osteosarcoma has been more and more reported [3–5]. In this study, we performed a systematic review and meta-analysis, to better understand the prognostic value of miRNAs in osteosarcoma. Using a systemic review and meta-analysis, this study comprehensively evaluated the prognostic value of miRNA expression in osteosarcoma.

## RESULTS

### Study characteristics

**Figure 1:**
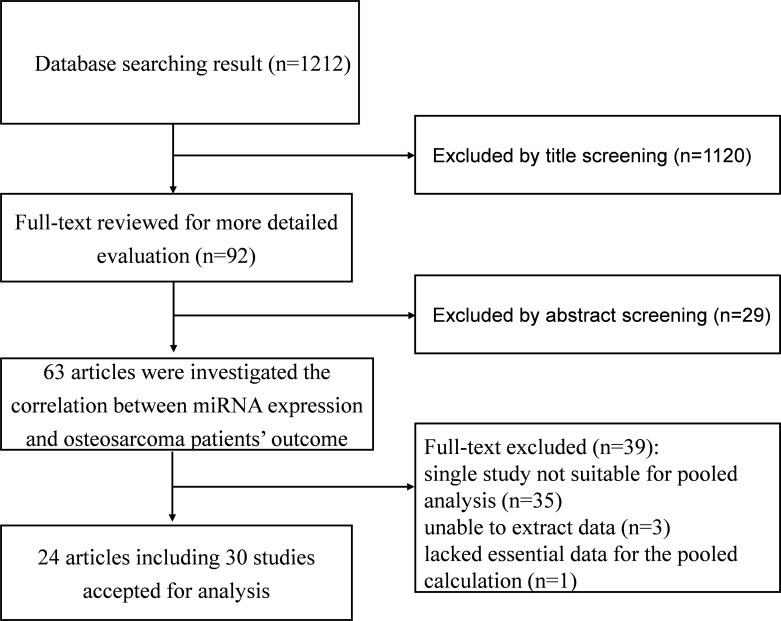
Flow diagram of the study selection process

A total of 1,212 references were initially retrieved using the search strategy. After screening the titles and abstracts, 63 references reported on the correlation between miRNA expression and either patient survival or disease recurrence in osteosarcoma. Among these, 35 studies reported at least one unique miRNA, so a pooled analysis would not be possible [6–40]. Finally, 24 articles encompassing 30 studies were include in the meta-analysis [3–5, 41–61] (Figure 1). The characteristics of the articles are shown in Table 1.

**Table 1:** The main characteristics of all studies in the meta-analysis

Study	country	Sample number	Tumor stage I/II/III/IV	MiRNAs identified	Follow-up months	Detected sample	Assay method	Cut-off value	Multivariate analysis	Survival analysis
Lu 2017 [3]	China	92	0/73/19/0	MiR-26a	over 60	tissue	qRT-PCR	median	NO	OS
Song 2014 [4]	China	144	IIA62/IIB-III82	MiR-26a	median 83	tissue	qRT-PCR	median	NO	OS, DFS
Sanchez-Diaz 2014 [5]	USA	27	NR	MiR-26a, miR-221, miR-126, miR-21, miR-92a	NR	tissue	qRT-PCR	median	YES	OS, RFS
Hong 2014 [41]	China	80	I-II50/III-IV30	MiR-29a/b/c	over 40	serum	qRT-PCR	median	NO	OS, DFS
Yang 2015 [42]	China	108	IIA63/IIB-III45	MiR-221	median 26.2	serum	qRT-PCR	median	YES	OS, RFS
Liu 2015 [43]	China	35	NR	MiR-214	NR	tissue	qRT-PCR	median	NO	OS
Wang 2014 [45]	China	92	NR	MiR-214	median 82	tissue	qRT-PCR	median	YES	OS, PFS
Allen-Rhoades 2015 [44]	USA	40	NR	MiR-214	NR	plasma	qRT-PCR	other	NO	OS
Liu 2015 [46]	China	122	I-II81/III-IV41	MiR-126	NR	tissue	qRT-PCR	median	YES	OS
Han 2015 [47]	China	107	IIA32/IIB-III75	MiR-195	median 42	tissue	qRT-PCR	median	YES	OS
Han 2015 [47]	China	99	IIA27/IIB-III72	MiR-195	NR	tissue	qRT-PCR	median	YES	OS
Cai 2015 [48]	China	166	IIA68/IIB-III98	MiR-195	median 87	serum	qRT-PCR	median	YES	OS
Han 2015 [49]	China	105	IIA46/IIB-III59	MiR-124	over 60	tissue	qRT-PCR	median	NO	OS
Wang 2016 [50]	China	69	6/48/15/0	MiR-124	over 20	tissue	qRT-PCR	other	NO	OS
Xu 2014 [51]	China	79	I-II39/III40	MiR-9	NR	tissue	qRT-PCR	median	NO	OS
Fei 2014 [52]	China	118	I-IIA64/IIB-III54	MiR-9	NR	serum	qRT-PCR	other	NO	OS
Yuan 2012 [53]	China	65	19/46/0/0	MiR-21	NR	serum	qRT-PCR	median	YES	OS
Ren 2016 [54]	China	84	I-IIA36/IIB-III48	MiR-21	median 86	tissue	qRT-PCR	median	YES	OS, DFS
Xu 2014 [55]	China	115	NR	MiR-382	over 60	tissue	qRT-PCR	median	NO	OS
Xu 2014 [56]	China	168	0/0/35/133	MiR-382	over 40	tissue	qRT-PCR	median	NO	MFS
Sarver 2013 [57]	USA	16	NR	MiR-382	NR	tissue	qRT-PCR	median	NO	OS, MFS
Ma 2014 [58]	China	89	I-III58/IV31	MiR-148a	median 41	blood	qRT-PCR	median	NO	OS, DFS
Zhang 2016 [59]	China	92	NR	MiR-148a	NR	tissue	qRT-PCR	median	NO	OS
Meng 2016 [60]	China	45	IIA27/IIB-III18	MiR-92a	median 36	serum	qRT-PCR	other	NO	OS
Wu 2010 [61]	China	42	2/36/4/0	MiR-21	mean 54.5	tissue	qRT-PCR	median	NO	OS

OS: overall survival; DFS: disease-specific survival; RFS: recurrence-free survival; MFS: metastasis-free survival.

### Quality assessment

Every eligible study enrolled in our meta-analysis was evaluated by the Newcastle-Ottawa Quality Assessment Scale (NOS). The NOS scores of every study range from 4 to 8, with an average of 6.54. The detailed information of NOS scores is shown in Table 2.

**Table 2:** Newcastle-Ottawa scale scores

Study	Selection	Comparability	Outcome	Total
Lu 2017 [3]	4	2	2 ^g^	8
Song 2014 [4]	4	1 ^f^	2 ^g^	7
Sanchez-Diaz 2014 [5]	4	0 ^e^ ^f^	1 ^h i^	5
Hong 2014 [41]	4	1 ^f^	2 ^g^	7
Yang 2015 [42]	4	1 ^f^	2 ^g^	7
Liu 2015 [43]	4	0 ^e^ ^f^	1 ^h i^	5
Wang 2014 [45]	4	0 ^e^ ^f^	3	7
Allen-Rhoades 2015 [44]	4	0 ^e^ ^f^	1 ^h i^	5
Liu 2015 [46]	4	0 ^e^ ^f^	0 ^g h i^	4
Han 2015 [47]	4	2	2 ^h^	8
Cai 2015 [48]	4	1 ^f^	3	8
Han 2015 [49]	4	1 ^f^	2 ^g^	7
Wang 2016 [50]	4	2	1 ^h i^	7
Xu 2014 [51]	4	1 ^f^	1 ^h i^	6
Fei 2014 [52]	4	2	1 ^h i^	7
Yuan 2012 [53]	4	1 ^f^	1 ^h i^	6
Ren 2016 [54]	4	1 ^f^	1 ^h i^	6
Xu 2014 [55]	4	1 ^e^	2 ^g^	7
Xu 2014 [56]	4	2	2 ^g^	8
Sarver 2013 [57]	4	0 ^e^ ^f^	0 ^g h i^	4
Ma 2014 [58]	4	2	2 ^g^	8
Zhang 2016 [59]	4	0 ^e^ ^f^	0 ^g h i^	4
Meng 2016 [60]	4	2	2 ^g^	8
Wu 2010 [61]	4	2	2 ^g^	8

Reasons for lost stars: ^a^ no description of the derivation of the cohort; ^b^ no description of the derivation of the non-exposed cohort; ^c^ no description of exposure ascertainment; ^d^ no description of whether outcome of interest was not present at the start of study; ^e^ study not controlling the most important factor such as TNM stage; ^f^ study not controlling other additional factors, such as age, gender, and smoke; ^g^ no description of outcome assessment; ^h^ inadequacy of follow-up of cohorts; ^I^ follow-up not long enough for outcomes to occur.

### miR-21

**Figure 2:**
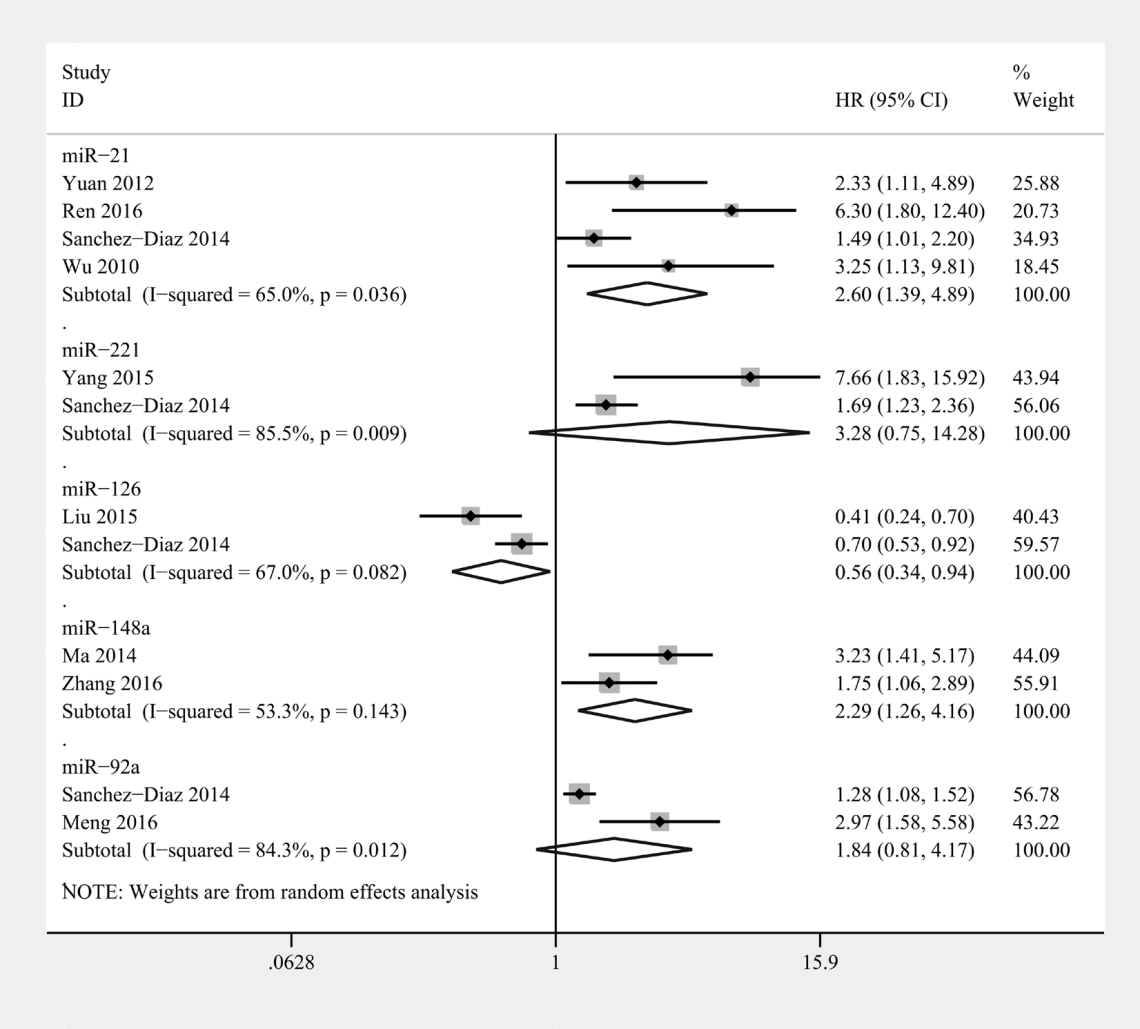
Forest plot of the relationship between overexpression miR-21, miR-221, miR-126, miR-148a, and miR-92a and overall survival (OS) in osteosarcoma patients with random-effects model

There were 4 studies assessing miR-21 as a predictor of survival in osteosarcoma using multivariate analysis, among which 3 used tumor tissues (*n* = 153) [5, 54, 61] and 1 used serum samples (*n* = 65) [53] (Table 3). Once pooled, studies with different baselines showed obvious statistical heterogeneity. Hence, we pooled the HRs using a random-effects model. The results showed that upregulated miR-21 was associated with worse overall survival (OS) in osteosarcoma (HR=2.60, 95% CI 1.39–4.89, *P* = 0.003) (Figure 2). Considering the inter-study heterogeneity, we excluded the serum miR-21 study and pooled the remaining tissue-based miR-21 studies. The results showed that elevated levels of miR-21 in tissue was associated with worse OS in patients with osteosarcoma (HR=2.88, 95% CI 1.12–7.38, *P* = 0.028). There was only one study that evaluated the correlation between miR-21 expression and disease-specific survival (DFS) in osteosarcoma, which reported that high levels of miR-21 were associated with poor DFS in osteosarcoma. Therefore, miR-21 may act as a marker of poor prognosis in patients with osteosarcoma.

**Table 3:** Summary of hazard ratios of miRNA expression in osteosarcoma

MiRNA	No. of studies	No. of patients	Survival outcome	Effects model	HR (95% CI)	*P* value	Heterogeneity	Reference
MiR-21	4	218	OS	Random	2.60 (1.39–4.89)	0.003*	*I*^2^ = 65.0%; *P* = 0.036	[5, 53, 54, 61]
MiR-214	3	166	OS	Fixed	3.81 (2.13–6.83)	< 0.001*	*I*^2^ = 42.8%; *P* = 0.174	[43–45]
MiR-382	2	171	OS	Fixed	0.51 (0.29–0.87)	0.013*	*I*^2^ = 16.6%; *P* = 0.274	[55, 57]
MiR-382	2	184	MFS	Fixed	0.45 (0.30–0.67)	< 0.001*	*I*^2^ = 21.0%; *P* = 0.261	[56, 57]
MiR-26a	2	236	OS	Fixed	0.43 (0.29–0.62)	< 0.001*	*I*^2^ = 0.0%; *P* = 0.876	[3, 4]
MiR-29a	2	127	OS	Fixed	2.66 (1.53–4.63)	0.001*	*I*^2^ = 46.0%; *P* = 0.174	[5, 41]
MiR-221	2	135	OS	Random	3.28 (0.76–14.28)	0.113	*I*^2^ = 85.5%; *P* = 0.009	[5, 42]
MiR-221	2	135	RFS	Random	2.92 (0.69–12.28)	0.144	*I*^2^ = 82.3%; *P* = 0.017	[5, 42]
MiR-126	2	149	OS	Random	0.56 (0.34–0.94)	0.029*	*I*^2^ = 67.0%; *P* = 0.082	[5, 46]
MiR-195	3	372	OS	Fixed	0.38 (0.27–0.54)	< 0.001*	*I*^2^ = 0.0%; *P* = 0.745	[47, 48]
MiR-124	2	174	OS	Fixed	0.34 (0.21–0.57)	< 0.001*	*I*^2^ = 0.0%; *P* = 0.810	[49, 50]
MiR-9	2	197	OS	Fixed	4.25 (3.10–5.84)	< 0.001*	*I*^2^ = 38.6%; *P* = 0.202	[51, 52]
MiR-148a	2	181	OS	Random	2.29 (1.26–4.16)	0.006*	*I*^2^ = 53.3%; *P* = 0.143	[58, 59]
MiR-92a	2	72	OS	Random	1.84 (0.81–4.17)	0.143	*I*^2^ = 84.3%; *P* = 0.012	[5, 60]

OS: overall survival; RFS: recurrence-free survival; MFS: metastasis-free survival.

### miR-214

**Figure 3:**
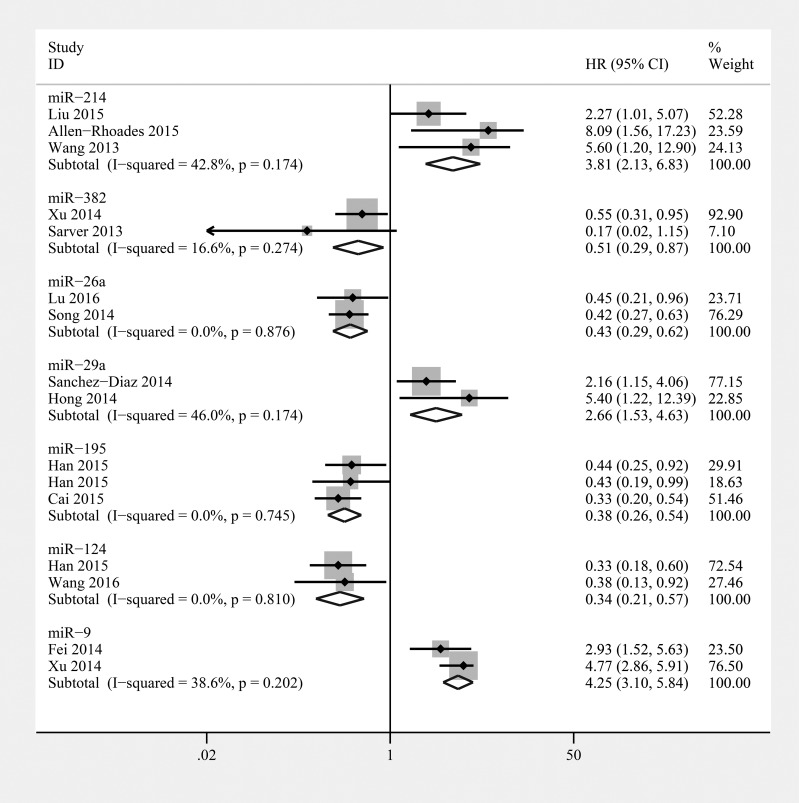
Forest plot of the relationship between overexpression miR-214, miR-382, miR-26a, miR-29a, miR-195, miR-124, and miR-9 and overall survival (OS) in osteosarcoma patients with fixed-effects model

There were 3 studies that assessed miR-214 as a predictor of survival in osteosarcoma using univariate analysis (*n* = 176), of which 2 used tumor tissues [43, 45] and 1 used plasma samples [44] (Table 3). Based on the 3 studies providing OS of patients, the pooled HR of 3.81 (95% CI 2.13–6.83, *P* < 0.001) indicated that elevated miR-214 was significantly associated with poor prognosis in osteosarcoma (Figure 3). In the subgroups that analyzed miR-214 in tumor tissue, we found that patients with high miR-214 expression in tissue had a shorter OS (HR = 3.02, 95% CI 1.55–5.88, *P* = 0.001). In addition, Wang et al. reported that upregulation of miR-214 was significantly associated with worse PFS in osteosarcoma. Therefore, high levels of miR-214 predict a worse clinical outcome.

### miR-382

**Figure 4:**
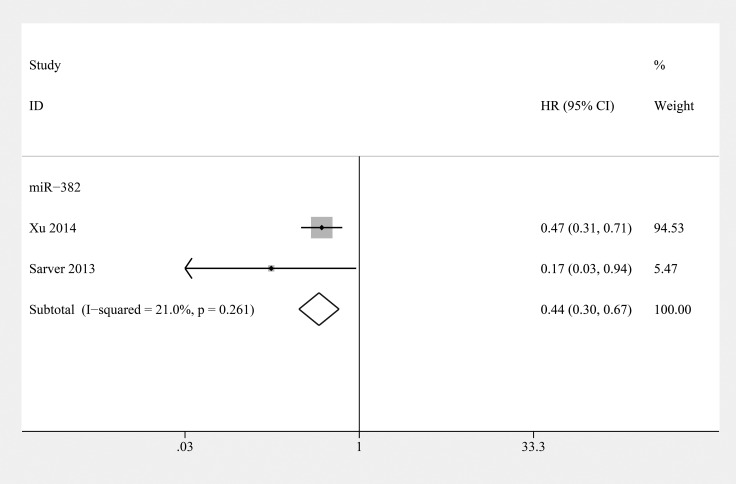
Forest plot of the relationship between overexpression miR-382 and metastasis-free survival (MFS) in osteosarcoma patients with fixed-effects model

Three studies showed that elevated miR-382 levels in tumor tissue were predictive of favorable OS and metastasis-free survival (MFS) in osteosarcoma as assessed using univariate analysis (*n* = 299) [55–57] (Table 3). We calculated a pooled HR for the correlation between elevated miR-382 expression and OS (HR: 0.51; 95% CI 0.29–0.87, *P* = 0.013) (Figure 3). In addition, the combined HR for MFS was 0.45 (95% CI 0.30–0.67, *P* < 0.001) (Figure 4). The pooled HRs for OS and MFS were consistent, and we can conclude that draw a conclusion that miR-382 indicated poor prognosis in osteosarcoma.

### miR-26a

Two studies described low levels of miR-26a in tumor tissue as a predictive biomarker for worse OS in osteosarcoma based on univariate analyses (*n* = 236) [3, 4] (Table 3). The combined HR showed that elevated levels of tissue-based miR-26a were significantly associated with a better OS outcome in osteosarcoma (HR: 0.43; 95% CI 0.29–0.62, *P* < 0.001) (Figure 3). Moreover, Song et al. reported that low miR-26a expression was associated with worse clinicopathological characteristics and DFS in osteosarcoma. Therefore, miR-26a may act as a tumor suppressor in osteosarcoma.

### miR-29a/b/c

Two studies reported that miR-29a overexpression is a poor prognostic marker in osteosarcoma based on multivariate analyses (*n* = 127) [5, 41] (Table 3). A combined HR of 2.66 (95% CI 1.53–4.63, *P* = 0.001) showed that miR-29a overexpression was significantly associated with poor OS in osteosarcoma (Figure 3). In addition, Hong et al. reported that miR-29b upregulation was also significantly associated with worse OS and DFS in osteosarcoma. However, the OS and DFS of osteosarcoma patients with high levels of miR-29c expression showed no significant differences from the OS and DFS of patients with low levels of miR-29c expression.

### miR-221

**Figure 5:**
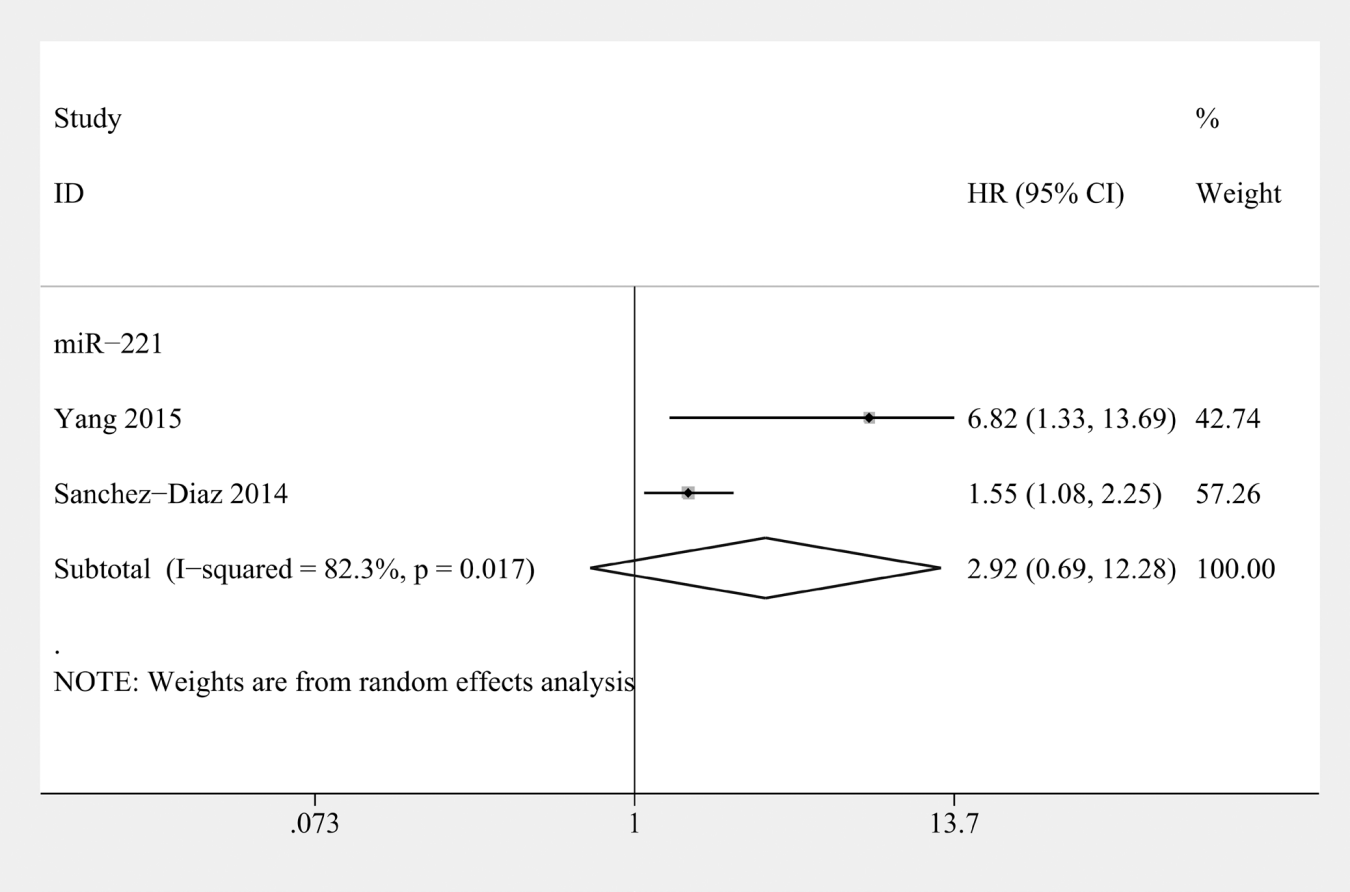
Forest plot of the relationship between overexpression miR-221 and recurrence-free survival (RFS) in osteosarcoma patients with random-effects model

Two studies assessed high miR-221 levels as a predictor of poor OS and recurrence-free survival (RFS) in osteosarcoma (*n* = 135) [5, 42] (Table 3). The baselines of the patients in these 2 studies were similar. The conclusions were reached using a multivariate analysis. The combined results showed that miR-221 expression was not associated with either worse OS (HR=3.28; 95% CI 0.76–14.28, *P* = 0.113) (Figure 2) or RFS (HR= 2.92; 95% CI 0.69–12.28, *P* = 0.144) (Figure 5) in osteosarcoma. Therefore, the prognostic value of miR-221 remains unclear.

### miR-126

Two studies reported that high miR-126 expression in tumor tissue is a favourable prognostic marker in osteosarcoma using multivariate analyses (*n* = 149) [5, 46] (Table 3). The combined HR indicated that elevated levels of miR-126 in tumor tissue were significantly associated with longer OS in osteosarcoma (HR: 0.56; 95% CI 0.34–0.94, *P* = 0.029) (Figure 2).

### miR-195

Two articles comprising 3 studies researched the relationship between miR-195 and prognosis in osteosarcoma (*n* = 372) [47, 48] (the study by Han et al. contained two independent cohorts [47]) (Table 3). A combined HR of 0.38 (95% CI 0.27–0.54, *P* = 0.001) showed that elevated miR-195 levels were significantly associated with poor OS in osteosarcoma (Figure 3).

### miR-124

Two studies reported that high miR-124 expression in tumor tissue is a favourable prognostic marker in osteosarcoma based on univariate analyses (*n* = 174) [49, 50] (Table 3). The baselines of the patients in these 2 studies were similar. miR-124 was measured in tissue, and the pooled estimate of risk was 0.34 (95% CI 0.21–0.57 *P* < 0.001) with no significant inter-study heterogeneity (*P* = 0.810, *I2*=0%) (Figure 3). Thus, miR-124 may act as a marker for increased OS in patients with osteosarcoma.

### miR-9

Two studies reported that elevated miR-9 levels are a predictive marker for worse OS in osteosarcoma (*n* = 197) [51, 52] (Table 3). The combined HR showed that elevated miR-9 levels were significantly associated with worse OS outcome in osteosarcoma (HR: 4.25; 95% CI 3.10–5.84, *P* < 0.001) (Figure 3).

### miR-148a

Two studies reported that high levels of miR-148a expression are a strong prognostic marker in osteosarcoma based on univariate analyses (*n* = 181) [58, 59] (Table 3). A combined HR of 2.29 (95% CI 1.26–4.16, *P* = 0.006) showed that miR-148a overexpression was significantly associated with poor OS in osteosarcoma (Figure 2). In addition, Ma et al. reported that miR-148a upregulation was also significantly associated with worse DFS in osteosarcoma. Therefore, miR-148a may play a role as an oncogene in osteosarcoma.

### miR-92a

Two studies reported that high levels of miR-92a are a poor prognostic marker in osteosarcoma (*n* = 72) [5, 60] (Table 3). The combined results showed that miR-92a expression was not associated with worse OS (HR=1.84; 95% CI 0.81–4.17, *P*=0.143) in osteosarcoma (Figure 2). Therefore, the prognostic value of miR-92a remains unclear.

### Publication bias

Publication bias was assessed using Egger's and Begg's tests. All the *P* values of Egger's and Begg's tests were bigger than 0.05, indicating that no significant publication bias was observed in our meta-analysis. We did not analyse the publication bias using funnel plots because of the number of included studies.

## DISCUSSION

Various chemotherapy regimens have significantly improved the survival of patients with osteosarcoma. However, patients receiving similar therapy usually presented outcomes, suggesting an urgent demand for reliable prognostic biomarkers. With the development of high-throughput array profiling, it is possible to obtain a more accurate prediction of patient prognosis in osteosarcoma based on the miRNA profile. In our study, our aim was to identify miRNAs that were correlate with the survival of patients with osteosarcoma, which could be used to inform treatment decisions and evaluate patient prognosis.

Kim et al. performed a previous meta-analysis assessing the prognostic value of miRNAs in osteosarcoma [62]. A total of 25 studies comprising 2,278 patients were included in that analysis. They concluded that decreased miRNA expression in tumor tissue is associated with worse outcome of patients with osteosarcoma. However, in our opinion, the results of their study are not convincing enough. In Kim's study, different miRNAs (miR-132, 145, 382, 133a, 26a, 340, 20a, 92a, 143, 451, 144, 22, 195, 124, 449a, 99a, 224, 210, 17–92 cluster, 128, 9, 214, 542-5p, 130b, 130a, 199b-5p) were pooled together for analysis. The function and targets of each miRNA are not the same, and the expression of the different miRNAs is highly varied. It is meaningless to simply combine the different miRNAs in a single meta-analysis, and the logic of that study is faulty. In our study, we revealed that high levels of miR-21, miR-214, miR-29, miR-9 and miR-148a were associated with worse outcomes in osteosarcoma, while miR-382, miR26a, miR-126, miR-195 and miR-124 expression showed the opposite results. We did not pool these miRNAs together.

Thus, our research represents the first focused systematic review and pooled analysis of prognostic miRNAs relevant to osteosarcoma. The results of our meta-analysis revealed that high levels of miR-21, miR-214, miR-29, miR-9 and miR-148a were associated with poor prognosis in osteosarcoma. Additionally, downregulated miR-382, miR26a, miR-126, miR-195 and miR-124 expression indicated poor prognosis in osteosarcoma.

miR-21 has often been reported to play as tumor oncogene in various types of tumors. In osteosarcoma, it modulates cell invasion and migration by directly targeting RECK and PTEN [63, 64]. In addition, miR-21 reduced the anti-tumor effect of cisplatin by regulating Bcl-2 expression in osteosarcoma cells [65]. miR-214 can function as either an oncogene or a tumor suppressor in various types of human cancer. In osteosarcoma, miR-214 promotes cell proliferation and invasion by regulating LZTS1 expression [66]. Elevated miR-214 levels promotes the progression of human osteosarcoma by regulating Wnt/β-catenin signaling pathway [67]. Knockdown of miR-29 induces apoptosis of osteosarcoma cells by regulating TGF-β1/PUMA signalling [68]. Upregulated miR-9 expression can increase cell proliferation, migration, and invasion as well as decrease the apoptotic ability of the cells [69]. miR-9 promotes cell growth by targeting the GCIP tumor suppressor in osteosarcoma [70]. Overexpression of miR-148a promotes osteosarcoma cell growth by targeting PTEN [59]. Thus, miR-21, miR-214, miR-9 and miR-148a function as onco-miRNAs in osteosarcoma.

miR-382 overexpression restrained osteosarcoma cell proliferation and chemoresistance by regulating HIPK3 and KLF12 [55]. miR-382 overexpression inhibited cancer stem cell-induced tumor formation by directly targetingYB-1, and the combination of miR-382 overexpression with doxorubicin treatment prevented disease relapse in osteosarcoma patients [56]. miR-26a inhibits tumor growth of osteosarcoma and the stem cell-like phenotype by targeting Jagged1 [3]. miR-26a could also inhibit the proliferative abilities of osteosarcoma by targeting IGF-1 [71]. miR-126 promotes apoptosis and inhibits proliferation in osteosarcoma cells without significantly effecting cell cycle arrest at G1 phase [72]. miR-126 overexpression in osteosarcoma cells inhibited cell growth and invasion by targeting Sox2 [73]. Overexpression of miR-195 inhibits cell growth and invasion in osteosarcoma cells by targeting CCND1 [47]. miR-195 overexpression inhibited cell invasion and cell growth in osteosarcoma cells by targeting FASN [74]. Overexpression of miR-124 could reduce osteosarcoma cell proliferation, invasion and migration as well as promote cell apoptosis [49]. miR-124 inhibited cell growth and invasion in osteosarcoma cells by targeting ROR2, SPHK1, Rac1 and B7-H3 [50, 75–77]. Thus, miR-382, miR26a, miR-126, miR-195 and miR-124 act as tumor suppressors in osteosarcoma, which is consistent with our results; thus, our conclusions are reliable and robust.

However, our study still has some limitations. First, some of the pooled analyses for miR-21, miR-221, miR-126, and miR-148a contained studies with significant statistical heterogeneity. Second, some HRs were extracted from the reported survival curves, which will inevitably lead to small statistical errors. Third, most of the articles included in this meta-analysis were from China. Consequently, the samples used in this study were imbalance. Finally, the cutoff value used in each study was different such that a clear threshold could not be established.

In conclusion, our pooled analysis revealed that high levels of miR-21, miR-214, miR-29, miR-9 and miR-148a were associated with poor prognosis in osteosarcoma, whereas reduced miR-382, miR26a, miR-126, miR-195 and miR-124 expression showed similar results. Further prospective multicentre that are adequately designed with a larger sample size are needed to confirm the prognostic value of this panel of miRNAs in osteosarcoma and to explore more effective therapeutic stategies. In addition, further comparative analysis among the different miRNAs should be made, to identify which find which miRNA would be the most effective marker to improving patient prognosis.

## MATERIALS AND METHODS

This systemic review and meta-analysis was performed following the Observational Studies in Epidemiology (MOOSE) guidelines [78].

### Search strategy

Literature searches were conducted using the PubMed, Embase, the Cochrane Library, China National Knowledge Infrastructure, and Wanfang data-bases (final search conducted January 1, 2017). The keywords combinations in the search strategy were “microRNA OR microRNAs OR miR OR miRNA” (all fields) AND “osteosarcomas OR osteosarcoma OR osteogenic sarcoma” (all fields) AND “prognosis OR prognostic OR survival” (all fields). Searches were limited to the English language publications.

### Inclusion and exclusion criteria

Eligible studies included in the systemic review and meta-analysis met the following criteria: (1) focused on patients with osteosarcoma; (2) assayed type either blood or tissue samples; (3) investigated the miRNA prognostic value, (4) clearly defined the cut-off, (5) clearly described the miRNA measurement method (Table 4). Studies were excluded if the met one of the following criteria (1) single study focused on a miRNA not investigated by another study, (2) unable to extract the data, (3) a lack of essential data for the pooled calculation.

**Table 4:** Criteria for the inclusion of prognostic miRNA studies

Study design	Prospective or retrospective cohort design with an appropriate study population
Tumor type	Osteosarcoma
Assay type	Tissue or blood
MiRNA measure	Quantitative real time reverse transcription PCR
Survival measure	OS or RFS or DFS or MFS
Analysis	Reporting of the HRs including 95% CIs or Kaplan Meier survival curves
Follow-up time	Any
Language	English or Chinese

OS: overall survival; DFS: disease-specific survival; RFS: recurrence-free survival; MFS: metastasis-free survival.

### Data extraction and quality assessment

The database search was independently reviewed by two authors (D. Cheng and X. Qiu). The essential information was independently by two investigators. If the statistical variables were not described in the study, we calculated from the available numerical data in the articles using the methods described by Tierney [79]. The quality of included studies was assessed by NOS according to the following categories: selection (description of the derivation of the cohort, description of the derivation of the non-exposed cohort, description of exposure ascertainment, description of whether outcome of interest was not present at the start of study), comparability (study controlled the most important factor, study controlled other additional factors), and outcome of interest (description of outcome assessment, adequacy of follow-up of cohorts, follow-up long enough for outcomes to occur) [80]. A total of nine items were extracted and each item scored 1. The total score of NOS ranged from 0 to 9, and we considered studies as high quality if they met at least six scores.

### Statistical analysis

An observed HR < 1 suggested a more favourable prognosis in patients with miRNA overexpression, and an HR > 1 indicated a worse prognosis. HRs and their 95% CIs were combined to measure the effective value of miRNA expression on prognosis. If the statistical data were described in the study, we extracted them directly. Otherwise, they were calculated from available numerical data in the articles according to the methods described by Tierney [79]. The data from Kaplan-Meier survival curves were read by Engauge Digitizer version 4.1, and three independent researchers read the curves to reduce reading variability. We also sent e-mail to the corresponding authors of eligible articles requesting additional information and original data needed for the meta-analysis. Statistical heterogeneity was assessed by calculating the *I*^2^ statistic, and assessing the *P* value [81, 82]. An *I*^2^ value exceeding 50% and/or the *P* value less than 0.05 indicated the presence of heterogeneity, and a random-effects model was used. Otherwise, the fixed-effects model was used. Publication bias was estimated using Begg's and Egger's tests. All analyses were performed using STATA vision 12.0 (Stata Corporation, College Station, TX, USA). A *P* value less than 0.05 was considered statistically significant except where otherwise specified.

## References

[1] Zhang J, Yan YG, Wang C, Zhang SJ, Yu XH, Wang WJ (2015). MicroRNAs in osteosarcoma. Clin Chim Acta.

[2] Siegel HJ, Pressey JG (2008). Current concepts on the surgical and medical management of osteosarcoma. Expert Rev Anticancer Ther.

[3] Lu J, Song G, Tang Q, Yin J, Zou C, Zhao Z, Xie X, Xu H, Huang G, Wang J, Lee DF, Khokha R, Yang H, Shen J (2017). MiR-26a inhibits stem cell-like phenotype and tumor growth of osteosarcoma by targeting Jagged1. Oncogene.

[4] Song QC, Shi ZB, Zhang YT, Ji L, Wang KZ, Duan DP, Dang XQ (2014). Downregulation of microRNA-26a is associated with metastatic potential and the poor prognosis of osteosarcoma patients. Oncol Rep.

[5] Sanchez-Diaz PC, Hsiao TH, Zou Y, Sugalski AJ, Heim-Hall J, Chen Y, Langevin AM, Hung JY (2014). In silico functional analyses and discovery of survival-associated microRNA signatures in pediatric osteosarcoma. Oncoscience.

[6] Li RZ, Wang LM (2016). Decreased microRNA-452 expression and its prognostic significance in human osteosarcoma. World J Surg Oncol.

[7] Lian D, Wang ZZ, Liu NS (2016). MicroRNA-1908 is a biomarker for poor prognosis in human osteosarcoma. Eur Rev Med Pharmacol Sci.

[8] Zhao J, Chen F, Zhou Q, Pan W, Wang X, Xu J, Ni L, Yang H (2016). Aberrant expression of microRNA-99a and its target gene mTOR associated with malignant progression and poor prognosis in patients with osteosarcoma. Onco Targets Ther.

[9] Zhou S, Wang B, Hu J, Zhou Y, Jiang M, Wu M, Qin L, Yang X (2016). miR-421 is a diagnostic and prognostic marker in patients with osteosarcoma. Tumour Biol.

[10] Song K, Liu N, Yang Y, Qiu X (2016). Regulation of osteosarcoma cell invasion through osteopontin modification by miR-4262. Tumour Biol.

[11] Liu S, Feng P (2015). MiR-203 Determines Poor Outcome and Suppresses Tumor Growth by Targeting TBK1 in Osteosarcoma. Cell Physiol Biochem.

[12] Cheng DD, Yu T, Hu T, Yao M, Fan CY, Yang QC (2015). MiR-542-5p is a negative prognostic factor and promotes osteosarcoma tumorigenesis by targeting HUWE1. Oncotarget.

[13] Wang NG, Wang DC, Tan BY, Wang F, Yuan ZN (2015). Down-regulation of microRNA152 is associated with the diagnosis and prognosis of patients with osteosarcoma. Int J Clin Exp Pathol.

[14] Yu LD, Jin RL, Gu PC, Ling ZH, Lin XJ, Du JY (2015). Clinical significance of microRNA-130b in osteosarcoma and in cell growth and invasion. Asian Pac J Trop Med.

[15] Wang T, Ji F, Dai Z, Xie Y, Yuan D (2015). Increased expression of microRNA-191 as a potential serum biomarker for diagnosis and prognosis in human osteosarcoma. Cancer Biomark.

[16] Zeng H, Zhang Z, Dai X, Chen Y, Ye J, Jin Z (2016). Increased Expression of microRNA-199b-5p Associates with Poor Prognosis Through Promoting Cell Proliferation, Invasion and Migration Abilities of Human Osteosarcoma. Pathol Oncol Res.

[17] Tang J, Zhao H, Cai H, Wu H (2015). Diagnostic and prognostic potentials of microRNA-27a in osteosarcoma. Biomed Pharmacother.

[18] Sun B, Yang M, Li M, Wang F (2015). The microRNA-217 functions as a tumor suppressor and is frequently downregulated in human osteosarcoma. Biomed Pharmacother.

[19] Wang G, Shen N, Cheng L, Lin J, Li K (2015). Downregulation of miR-22 acts as an unfavorable prognostic biomarker in osteosarcoma. Tumour Biol.

[20] Wang W, Zhou X, Wei M (2015). MicroRNA-144 suppresses osteosarcoma growth and metastasis by targeting ROCK1 and ROCK2. Oncotarget.

[21] Wang Y, Jia LS, Yuan W, Wu Z, Wang HB, Xu T, Sun JC, Cheng KF, Shi JG (2015). Low miR-34a and miR-192 are associated with unfavorable prognosis in patients suffering from osteosarcoma. Am J Transl Res.

[22] Zhou J, Wu S, Chen Y, Zhao J, Zhang K, Wang J, Chen S (2015). microRNA-143 is associated with the survival of ALDH1+CD133+ osteosarcoma cells and the chemoresistance of osteosarcoma. Exp Biol Med (Maywood).

[23] Chong Y, Zhang J, Guo X, Li G, Zhang S, Li C, Jiao Z, Shao M (2014). MicroRNA-503 acts as a tumor suppressor in osteosarcoma by targeting L1CAM. PLoS One.

[24] Tian R, Xie X, Han J, Luo C, Yong B, Peng H, Shen J, Peng T (2014). miR-199a-3p negatively regulates the progression of osteosarcoma through targeting AXL. Am J Cancer Res.

[25] Zhang F, Huang W, Sheng M, Liu T (2015). MiR-451 inhibits cell growth and invasion by targeting CXCL16 and is associated with prognosis of osteosarcoma patients. Tumour Biol.

[26] Chen J, Zhou J, Chen X, Yang B, Wang D, Yang P, He X, Li H (2015). miRNA-449a is downregulated in osteosarcoma and promotes cell apoptosis by targeting BCL2. Tumour Biol.

[27] Zhang C, Yao C, Li H, Wang G, He X (2014). Serum levels of microRNA-133b and microRNA-206 expression predict prognosis in patients with osteosarcoma. Int J Clin Exp Pathol.

[28] Cai H, Lin L, Tang M, Wang Z (2014). Combined microRNA-340 and ROCK1 mRNA profiling predicts tumor progression and prognosis in pediatric osteosarcoma. Int J Mol Sci.

[29] Tang M, Lin L, Cai H, Tang J, Zhou Z (2013). MicroRNA-145 downregulation associates with advanced tumor progression and poor prognosis in patients suffering osteosarcoma. Onco Targets Ther.

[30] Yang J, Gao T, Tang J, Cai H, Lin L, Fu S (2013). Loss of microRNA-132 predicts poor prognosis in patients with primary osteosarcoma. Mol Cell Biochem.

[31] Ji F, Zhang H, Wang Y, Li M, Xu W, Kang Y, Wang Z, Cheng P, Tong D, Li C, Tang H (2013). MicroRNA-133a, downregulated in osteosarcoma, suppresses proliferation and promotes apoptosis by targeting Bcl-xL and Mcl-1. Bone.

[32] Cai H, Lin L, Tang M, Wang Z (2013). Prognostic evaluation of microRNA-210 expression in pediatric osteosarcoma. Med Oncol.

[33] Niu J, Sun Y, Guo Q, Niu D, Liu B (2016). Serum miR-95-3p is a diagnostic and prognostic marker for osteosarcoma. Springerplus.

[34] Liu JD, Xin Q, Tao CS, Sun PF, Xu P, Wu B, Qu L, Li SZ (2016). Serum miR-300 as a diagnostic and prognostic biomarker in osteosarcoma. Oncol Lett.

[35] Pang PC, Shi XY, Huang WL, Sun K (2016). miR-497 as a potential serum biomarker for the diagnosis and prognosis of osteosarcoma. Eur Rev Med Pharmacol Sci.

[36] Cao L, Wang J, Wang PQ (2016). MiR-326 is a diagnostic biomarker and regulates cell survival and apoptosis by targeting Bcl-2 in osteosarcoma. Biomed Pharmacother.

[37] Zhang C, Yao C, Li H, Wang G, He X (2014). Combined elevation of microRNA-196a and microRNA-196b in sera predicts unfavorable prognosis in patients with osteosarcomas. Int J Mol Sci.

[38] Zhou Z, Wang Z, Wei H, Wu S, Wang X, Xiao J (2016). Promotion of tumour proliferation, migration and invasion by miR-92b in targeting RECK in osteosarcoma. Clin Sci (Lond).

[39] Zhang H, Yin Z, Ning K, Wang L, Guo R, Ji Z (2014). Prognostic value of microRNA-223/epithelial cell transforming sequence 2 signaling in patients with osteosarcoma. Hum Pathol.

[40] Dong J, Liu Y, Liao W, Liu R, Shi P, Wang L (2016). miRNA-223 is a potential diagnostic and prognostic marker for osteosarcoma. J Bone Oncol.

[41] Hong Q, Fang J, Pang Y, Zheng J (2014). Prognostic value of the microRNA-29 family in patients with primary osteosarcomas. Med Oncol.

[42] Yang Z, Zhang Y, Zhang X, Zhang M, Liu H, Zhang S, Qi B, Sun X (2015). Serum microRNA-221 functions as a potential diagnostic and prognostic marker for patients with osteosarcoma. Biomed Pharmacother.

[43] Liu CJ, Yu KL, Liu GL, Tian DH (2015). MiR214 promotes osteosarcoma tumor growth and metastasis by decreasing the expression of PTEN. Mol Med Rep.

[44] Allen-Rhoades W, Kurenbekova L, Satterfield L, Parikh N, Fuja D, Shuck RL, Rainusso N, Trucco M, Barkauskas DA, Jo E, Ahern C, Hilsenbeck S, Donehower LA, Yustein JT (2015). Cross-species identification of a plasma microRNA signature for detection, therapeutic monitoring, and prognosis in osteosarcoma. Cancer Med.

[45] Wang Z, Cai H, Lin L, Tang M (2014). Upregulated expression of microRNA-214 is linked to tumor progression and adverse prognosis in pediatric osteosarcoma. Pediatr Blood Cancer.

[46] Liu W, Zhao ZY, Shi L, Yuan WD (2015). Tissue microRNA-126 expression level predicts outcome in human osteosarcoma. Diagn Pathol.

[47] Han K, Chen X, Bian N, Ma B, Yang T, Cai C, Fan Q, Zhou Y, Zhao TB (2015). MicroRNA profiling identifies MiR-195 suppresses osteosarcoma cell metastasis by targeting CCND1. Oncotarget.

[48] Cai H, Zhao H, Tang J, Wu H (2015). Serum miR-195 is a diagnostic and prognostic marker for osteosarcoma. J Surg Res.

[49] Han G, Wang Y, Bi W, Jia J, Wang W (2015). MicroRNA-124 functions as a tumor suppressor and indicates prognosis in human osteosarcoma. Exp Ther Med.

[50] Wang L, Kang FB, Sun N, Wang J, Chen W, Li D, Shan BE (2016). The tumor suppressor miR-124 inhibits cell proliferation and invasion by targeting B7-H3 in osteosarcoma. Tumour Biol.

[51] Xu SH, Yang YL, Han SM, Wu ZH (2014). MicroRNA-9 expression is a prognostic biomarker in patients with osteosarcoma. World J Surg Oncol.

[52] Fei D, Li Y, Zhao D, Zhao K, Dai L, Gao Z (2014). Serum miR-9 as a prognostic biomarker in patients with osteosarcoma. J Int Med Res.

[53] Yuan J, Chen L, Chen X, Sun W, Zhou X (2012). Identification of serum microRNA-21 as a biomarker for chemosensitivity and prognosis in human osteosarcoma. J Int Med Res.

[54] Ren X, Shen Y, Zheng S, Liu J, Jiang X (2016). miR-21 predicts poor prognosis in patients with osteosarcoma. Br J Biomed Sci.

[55] Xu M, Jin H, Xu CX, Sun B, Mao Z, Bi WZ, Wang Y (2014). miR-382 inhibits tumor growth and enhance chemosensitivity in osteosarcoma. Oncotarget.

[56] Xu M, Jin H, Xu CX, Sun B, Song ZG, Bi WZ, Wang Y (2015). miR-382 inhibits osteosarcoma metastasis and relapse by targeting Y box-binding protein 1. Mol Ther.

[57] Sarver AL, Thayanithy V, Scott MC, Cleton-Jansen AM, Hogendoorn PC, Modiano JF, Subramanian S (2013). MicroRNAs at the human 14q32 locus have prognostic significance in osteosarcoma. Orphanet J Rare Dis.

[58] Ma W, Zhang X, Chai J, Chen P, Ren P, Gong M (2014). Circulating miR-148a is a significant diagnostic and prognostic biomarker for patients with osteosarcoma. Tumour Biol.

[59] Zhang H, Wang Y, Xu T, Li C, Wu J, He Q, Wang G, Ding C, Liu K, Tang H, Ji F (2016). Increased expression of microRNA-148a in osteosarcoma promotes cancer cell growth by targeting PTEN. Oncol Lett.

[60] Meng W, Zhang L, Li J, Wei M (2016). Correlation of miR-92a in plasma with prognosis in osteosarcoma. China Oncology.

[61] Wu Z, Liu X, Yang S (2010). Expression of miR-21 in osteosarcoma and its clinical significance. Orthopedic Journal of China.

[62] Kim YH, Goh TS, Lee CS, Oh SO, Kim JI, Jeung SH, Pak K (2017). Prognostic value of microRNAs in osteosarcoma: A meta-analysis. Oncotarget.

[63] Ziyan W, Shuhua Y, Xiufang W, Xiaoyun L (2011). MicroRNA-21 is involved in osteosarcoma cell invasion and migration. Med Oncol.

[64] Lv C, Hao Y, Tu G (2016). MicroRNA-21 promotes proliferation, invasion and suppresses apoptosis in human osteosarcoma line MG63 through PTEN/Akt pathway. Tumour Biol.

[65] Ziyan W, Yang L (2016). MicroRNA-21 regulates the sensitivity to cisplatin in a human osteosarcoma cell line. Ir J Med Sci.

[66] Xu Z, Wang T (2014). miR-214 promotes the proliferation and invasion of osteosarcoma cells through direct suppression of LZTS1. Biochem Biophys Res Commun.

[67] Zhu XB, Zhang ZC, Han GS, Han JZ, Qiu DP (2017). Overexpression of miR214 promotes the progression of human osteosarcoma by regulating the Wnt/betacatenin signaling pathway. Mol Med Rep.

[68] Wang CY, Ren JB, Liu M, Yu L (2016). Targeting miR-29 induces apoptosis of osteosarcoma MG-63 cells via regulation of TGF-beta1/PUMA signal. Eur Rev Med Pharmacol Sci.

[69] Qi XJ, Wang JF, Wang GD, Xu Q, Sun HL (2016). Pivotal role of microRNA-9 in osteosarcoma tumorigenesis and tumor progression. Genet Mol Res.

[70] Zhu SW, Li JP, Ma XL, Ma JX, Yang Y, Chen Y, Liu W (2015). miR-9 Modulates Osteosarcoma Cell Growth by Targeting the GCIP Tumor Suppressor. Asian Pac J Cancer Prev.

[71] Tan X, Fan S, Wu W, Zhang Y (2015). MicroRNA-26a inhibits osteosarcoma cell proliferation by targeting IGF-1. Bone Res.

[72] Jiang L, Tao C, He A, He X (2014). Overexpression of miR-126 sensitizes osteosarcoma cells to apoptosis induced by epigallocatechin-3-gallate. World J Surg Oncol.

[73] Yang C, Hou C, Zhang H, Wang D, Ma Y, Zhang Y, Xu X, Bi Z, Geng S (2013). miR-126 functions as a tumor suppressor in osteosarcoma by targeting Sox2. Int J Mol Sci.

[74] Mao JH, Zhou RP, Peng AF, Liu ZL, Huang SH, Long XH, Shu Y (2012). microRNA-195 suppresses osteosarcoma cell invasion and migration in vitro by targeting FASN. Oncol Lett.

[75] Zhang C, Hu Y, Wan J, He H (2015). MicroRNA-124 suppresses the migration and invasion of osteosarcoma cells via targeting ROR2-mediated non-canonical Wnt signaling. Oncol Rep.

[76] Zhou Y, Han Y, Zhang Z, Shi Z, Zhou L, Liu X, Jia X (2017). MicroRNA-124 upregulation inhibits proliferation and invasion of osteosarcoma cells by targeting sphingosine kinase 1. Hum Cell.

[77] Geng S, Zhang X, Chen J, Liu X, Zhang H, Xu X, Ma Y, Li B, Zhang Y, Bi Z, Yang C (2014). The tumor suppressor role of miR-124 in osteosarcoma. PLoS One.

[78] Stroup DF, Berlin JA, Morton SC, Olkin I, Williamson GD, Rennie D, Moher D, Becker BJ, Sipe TA, Thacker SB (2000). Meta-analysis of observational studies in epidemiology: a proposal for reporting. Meta-analysis Of Observational Studies in Epidemiology (MOOSE) group. JAMA.

[79] Tierney JF, Stewart LA, Ghersi D, Burdett S, Sydes MR (2007). Practical methods for incorporating summary time-to-event data into meta-analysis. Trials.

[80] Maxwell L, Santesso N, Tugwell PS, Wells GA, Judd M, Buchbinder R (2006). Method guidelines for Cochrane Musculoskeletal Group systematic reviews. J Rheumatol.

[81] Higgins JP, Thompson SG (2002). Quantifying heterogeneity in a meta-analysis. Stat Med.

[82] Higgins JP, Thompson SG, Deeks JJ, Altman DG (2003). Measuring inconsistency in meta-analyses. BMJ.

